# Supplementary figures and images for: The influence of the larval microbiome on susceptibility to Zika virus is mosquito genotype-dependent
Source: PLoS Pathog. 2023 Oct 30;19(10):e1011727. doi: 10.1371/journal.ppat.1011727 (PMC10635568; doi:10.1371/journal.ppat.1011727)

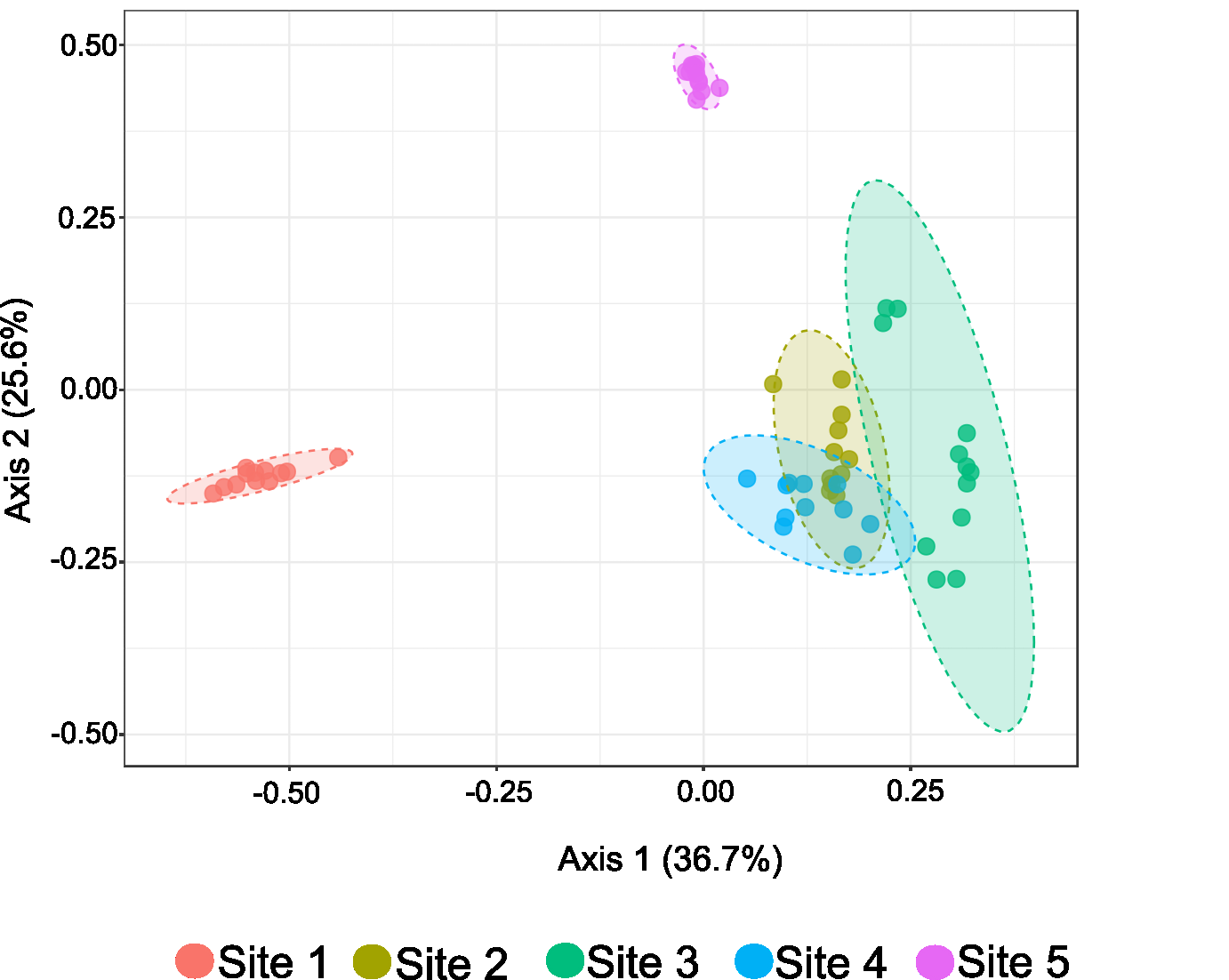

Supplement: S1 Fig — Structure of bacterial communities was determined by deep sequencing the V3-V4 region of the 16S gene in individual larvae collected from large metal drums at five sites in Senegal (Site1-Site5). Bacterial structure is represented by PCoA of a Bray-Curtis dissimilarity matrix based on mean genera abundance (PERMANOVA p = 0.001). (TIF) [file ppat.1011727.s001.tif]

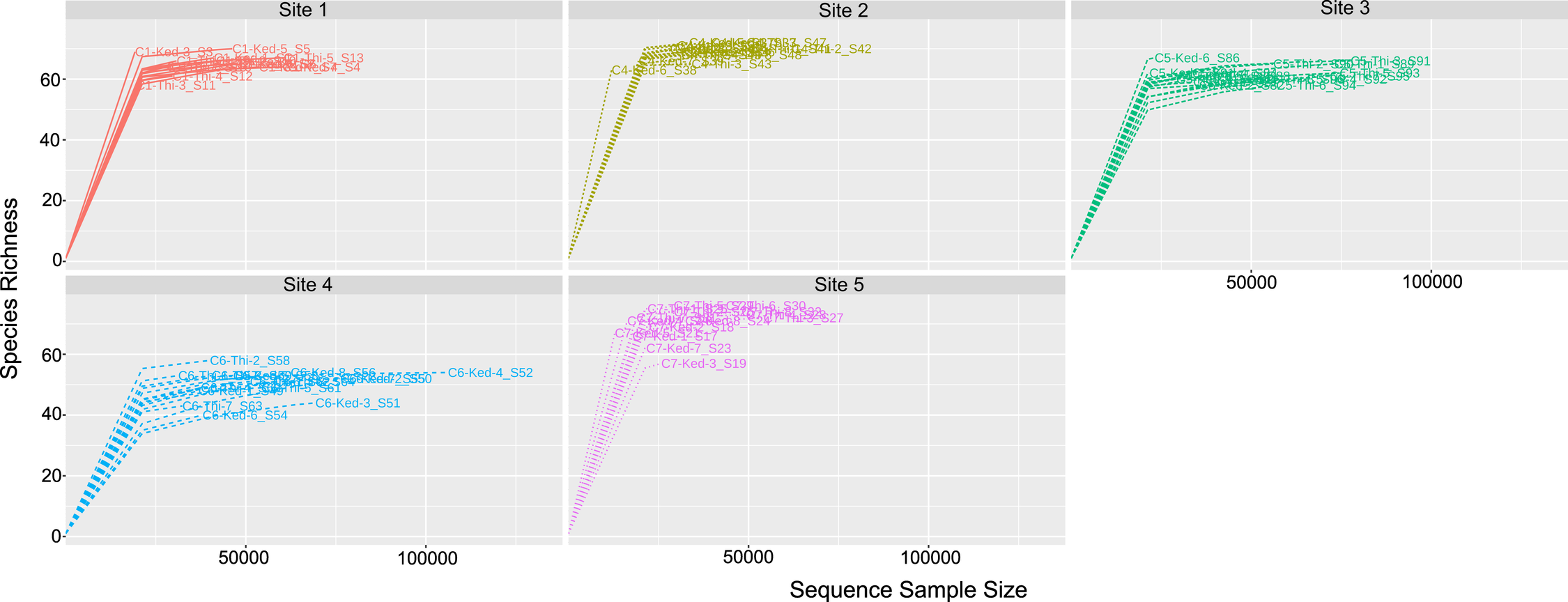

Supplement: S2 Fig — The number of species is shown on the Y axis, and the number of sequencing reads is shown on the X axis. (TIF) [file ppat.1011727.s002.tif]

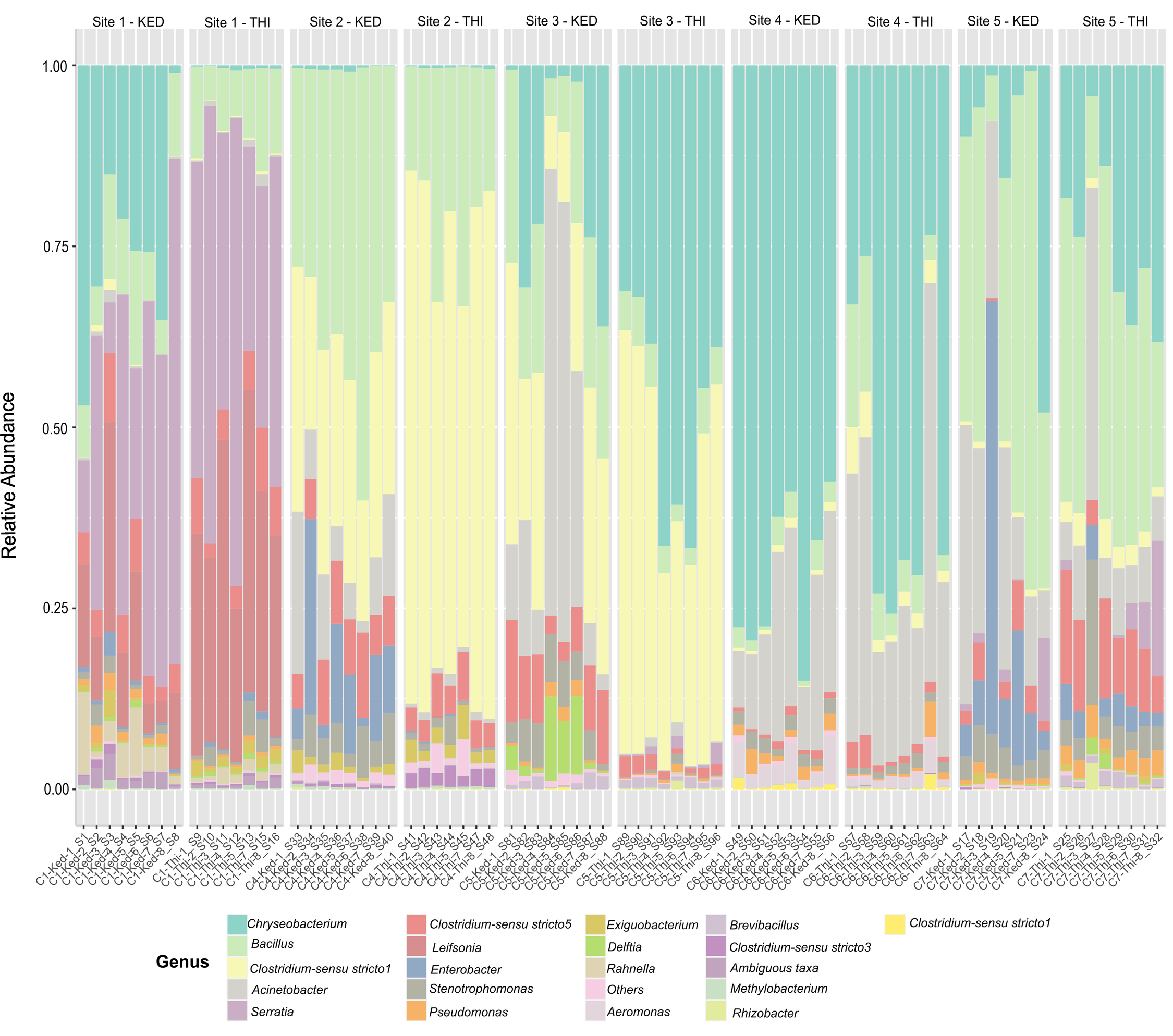

Supplement: S3 Fig — (TIF) [file ppat.1011727.s003.tif]

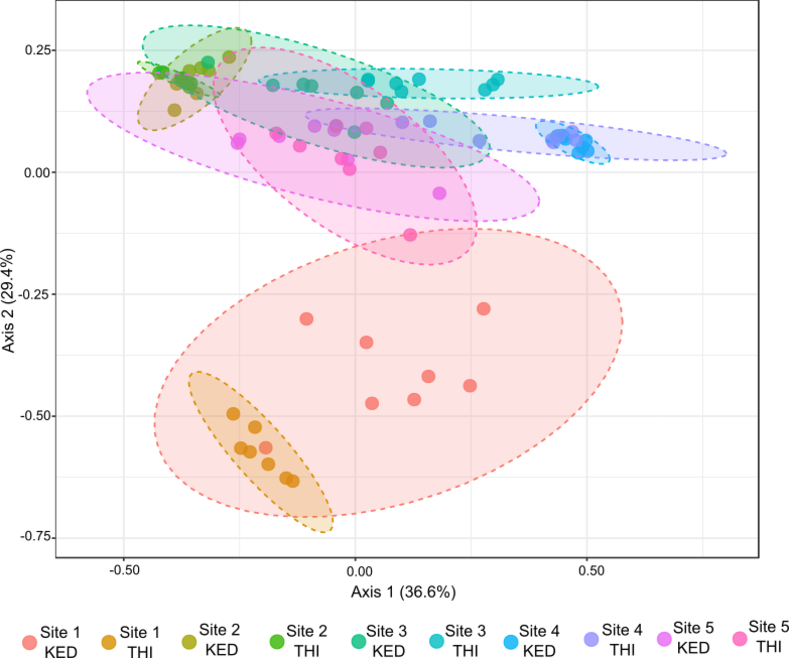

Supplement: S4 Fig — (TIF) [file ppat.1011727.s004.tif]
